# Native C3 is activated without proteolytic cleavage by transformation to C3(H_2_O) on phospholipid-scrambled cell membranes

**DOI:** 10.3389/fimmu.2025.1729532

**Published:** 2025-12-16

**Authors:** David Eikrem, Vivek A. Manivel, Jacob Whittaker, Osama A. Hamad, Camilla Mohlin, Anna Adler, Claudia Dührkop, Roland Ebert, Yuji Teramura, Kristina N. Ekdahl, Karin Fromell, Bo Nilsson

**Affiliations:** 1 Department of Immunology, Genetics and Pathology, Uppsala University, Uppsala, Sweden; 2 Department of Molecular Sciences, Swedish University of Agricultural Sciences, Uppsala, Sweden; 3 Linnaeus Centre for Biomaterials Chemistry, Linnaeus University, Kalmar, Sweden; 4 Cellular and Molecular Biotechnology Research Institute (CMB), National Institute of Advanced Industrial Science and Technology (AIST), Tsukuba, Ibaraki, Japan; 5 Master’s/Doctoral Program in Life Science Innovation (T-LSI), University of Tsukuba, Tsukuba, Ibaraki, Japan

**Keywords:** native C3, C3(H2O), alternative pathway activation, contact activation, non-proteolytical, phospholipid-scrambled cell membranes

## Abstract

Tick-over of C3 to fluid-phase C3(H_2_O) is considered the initiator of the alternative pathway by mediating random depositing of C3b on target surfaces. This slow mechanism does not explain the specificity and rapid activation of the alternative pathway *in vivo*. In thromboinflammatory disorders, C3(H_2_O) also exists in a bound form on activated platelets and endothelial cells. Here, we investigate this binding mechanism. C3, C3b, and C3(H_2_O) were exposed to activated platelets expressing phospholipid-scrambled membranes. Native C3 demonstrated the highest binding to activated platelets compared to C3b and soluble C3(H_2_O) and revealed the most efficient convertase (C3bBb) formation. The specific binding of annexin V to phospholipid-scrambled membranes, inhibited C3 binding to activated platelets, and to apoptotic PMN and endothelial cells, while properdin enhanced both binding and convertase generation. Model liposomes exposing phosphatidylserine, bound native C3 in a cholesterol-dependent manner. Neoepitopes and cryo-TEM, showed that the conformation of liposome-bound C3 resembles C3(H_2_O) and quartz crystal microbalance with dissipation monitoring (QCM-D) its ability to form C3bBb convertases. Thus, native C3 transforms into C3(H_2_O) by binding to phospholipid-scrambled membranes, identifying native C3 without proteolytic cleavage, as a direct recognition molecule of altered self (in this case exposure of phospholipids that are not found on the surface of native, healthy, cells) acting as a key initiator of the alternative pathway and a mediator of phagocytosis in thromboinflammatory pathologies.

## Graphical Abstract

**Figure:**
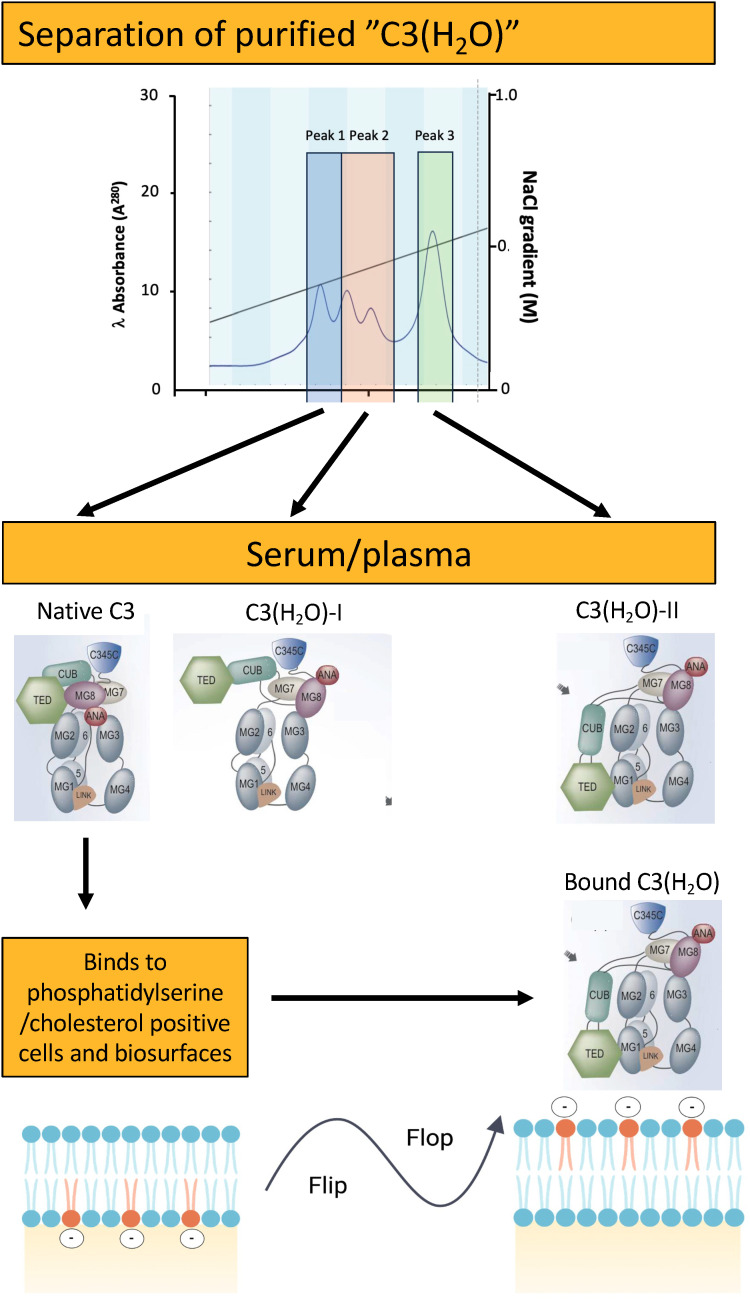

## Introduction

The alternative pathway (AP) of complement is initiated by formation of a complex between C3b and factor B (FB) in the presence of Mg^2+^, as reviewed in (1, 2). The AP is dependent on high concentrations of the individual components reflected in that, C3 and FB, are present in blood plasma at concentrations of 1 and 0.5 mg/mL, respectively (3). This initial complex formation is followed by cleavage of FB by factor D (FD) into Ba and Bb, to form the active enzymatic complex C3bBb. C3bBb is the AP C3 convertase and has a half-life of 90s (4, 5). The C3bBb convertase cleaves native C3 molecules into C3a and C3b. The C3b molecules trigger a positive feedback loop reaction, in which the generated C3b molecules form new AP convertases. However, the mechanism(s) how this is brought about has remained an enigma (6).

Initially, Lachmann put forward the tick-over theory as the explanation for this slow but constant-rate generation of C3b (7, 8). The tick-over theory states that low amounts of C3b are continuously generated to initiate an AP fluid-phase convertase. In the 1980s, Pangburn and co-workers described the spontaneous and continuous hydrolysis of the internal thiol ester in C3, generating a “C3b-like” molecule but with an intact α-chain, referred to as C3(H_2_O). In a recent study, we show that C3(H_2_O) is formed in plasma at a rate of approximately 0.2-0.4% per hour (9). C3(H_2_O) was demonstrated to have the ability to generate an active soluble AP C3 convertase, C3(H_2_O)Bb, which then activates the AP by cleaving native C3 molecules into C3a and C3b. In contrast to C3b, C3(H_2_O) lacks hemolytic activity (10, 11). Based on these findings, the hydrolysis of native C3 to C3(H_2_O) has been the predominant explanation to how the tick-over is brought about and to the activation of AP.

The present view of AP activation is that a few C3(H_2_O) molecules mediate random deposition of C3b that triggers an amplification loop on target surfaces. The fate of the activation is dependent on the regulation mediated by the surface which includes membrane-bound complement regulators and binding of soluble factor H (FH) and properdin to the surface (12). This mechanism is challenged by the rapid activation of the AP *in vivo*, which contradicts that the initiation of the AP depends solely on a few fluid-phase C3(H_2_O) molecules. To avoid a prolong lag phase before the activation takes off, a significant amount of initial C3b (or C3(H_2_O)) molecules bound to the target surface is needed to speed up the activation (13, 14). Deposition of large amounts of C3b can be achieved by activation of the classical and the lectin pathways, but in the absence of bound C3b molecules, the lag phase is significantly prolonged since the C3b generation is exponential, starting from a single nidus molecule (15). This was early recognized on cell surfaces (16) and is very well illustrated on non-regulated biomaterial surfaces both *in vitro* and *in vivo* where the lag phase may last up to 5–10 min (13, 14). Our previous studies also indicate that fluid-phase C3(H_2_O) is not an efficient partner in the AP convertase and is therefore less likely to be the primary contributor of C3b-like activity in the fluid-phase tick-over mechanism.

Adsorption of native C3 to microtiter plates shows that the conformation of C3 is changed when the protein binds to plastic surfaces. This is reflected in exposure of neoepitopes of e.g. monoclonal antibodies (mAbs), 4SD17.3, 7D84.1, and 7D169.1, which are linked to conformations of cell-bound C3b and C3 fragments and which are not exposed in fluid phase (14, 17–19). Also, many C3b-like functional properties appear, such as binding to complement receptor 1 (CR1, CD35), CR2 (CD21), and FH (20, 21).

In previous publications, we and others have shown that activated platelets, endothelial cells and liposomes bind non-proteolytically cleaved C3 to the surface, which has been interpreted as binding of preformed C3(H_2_O) (22–25). The binding of C3 to platelets is not inhibited by substances that inhibit complement activation, e.g., anti-C1q, compstatin, or in the presence of EDTA that chelated Ca^2+^ and Mg^2+^ (22). Despite that its binding is not the result of proteolytic activation, platelet-bound C3 has been shown to bind to complement receptors CR1 (CD35) and CR3 (CD11b/CD18), which facilitates platelet/leukocyte complex formation (22, 26).

In the present study we show that it is not preformed C3(H_2_O) but native C3 that binds to phospholipid-scrambled membranes e.g. on activated platelets and apoptotic cells. The binding requires negatively charged phospholipids e.g. phosphatidyl serine, and cholesterol and transforms the protein into a C3(H_2_O)-like molecule with similar conformational changes (**Graphical Abstract**). This mechanism of activation demonstrates that C3 is a specific recognition molecule of altered self (in this study focusing on exposure of phospholipids that are not found on the surface of native, healthy, cells) a key property of innate immunity. It gives C3 C3b-like properties with the ability to act as a ligand for phagocytic receptors and initiate the AP.

## Materials and methods

### Complement components and antibodies

C3 was prepared as described in (27). Other complement components were purchased from Complement Technology Inc., Tx, US. C3(H_2_O) was prepared by repeated freeze-thawing of C3 (C3(FT)) (28) or by treating native C3 with 0.2M methylamine (C3(met), treatments which both lead to disruption of the thiolester and loss of hemolytic activity (9). The anti-C3 monoclonal antibodies (mAb) 4SD17.3, 7D84.1, 7D169.1, and 7D398.1 against different neoepitopes in C3 (the C3(D) cluster) were produced according to Nilsson et al. (29).

### Separation of various forms of C3(H_2_O)

C3(H_2_O) generated by repeated freeze-thaw cycles (C3(FT) was separated by cation-exchange chromatography into native C3 and two different forms of C3(H_2_O) identified in (9). Two serum preparations were also separated: the initial serum immediately after preparation and the second after 24 h incubation at 37°C. C3 (H_2_O) (500 µg) or human serum (1 mL) were added to a MonoS 5/50 GL column. The rate was 0.5 mL/min with a gradient from 0 to 1 M NaCl in 20 mM Phosphate buffer pH 6.8. The chromatography was performed at room temperature (RT) (purified C3) or at +4°C (serum). Serum preparations had 1 mM benzamidine-HCl added after clotting for 45 minutes while collected fractions after separation had 1 mM phenylmethanesulfonyl fluoride (PMSF) added.

### C3 neoepitope ELISA

Sandwich ELISAs used the mAbs raised against C3 neoepitopes of the C3(D) epitope cluster (4SD17.3, 7D84.1, 7D169.1, and 7D398.1) (30) as capture antibodies (1-2 µg/mL) and biotinylated polyclonal (pAb) rabbit anti-C3c (10 µg/mL) followed by streptavidin-HRP (SA-HRP) from Cytiva Sweden AB (1:500) for detection.

A quantitative ELISA assessed the total amount of C3. It used rabbit anti-C3c pAb as both capture (3 µg/mL) and detection (biotinylated 3 µg/mL, followed by streptavin-HRP) as described above. The generation of C3a in supernatants was measured using a previously described C3a ELISA (31).

### Platelet isolation and activation

Blood anticoagulated with lepirudin (50 μg/mL; Schering AG, Saksa, Germany) or K2 EDTA (Greiner Bio-One, GmbH, Austria) was drawn from healthy volunteers who had been free from medication for at least 2 weeks prior to the donation. Platelet rich plasma (PRP) was prepared from whole blood by centrifugation at 150xg for 15 min at RT.

To isolate the platelets from plasma proteins, platelets were pelleted from PRP by centrifugation at 1100xg for 10 min and washed three times in Tyrode’s buffer (pH 6.5) (137 mM NaCl, 2.7 mM KCl, 1 mM MgCl_2_, 0.36 mM NaH_2_PO_4_, 12 mM NaHCO_3_, 2 mM CaCl_2_, 5.5 mM glucose) containing 3.5 mg/mL bovine serum albumin (BSA), 1 μM PGE1 (both from Sigma Aldrich, St Louis, MO, USA), and 50 μg/mL lepirudin. After being washed, the platelets were pelleted and resuspended in Tyrode’s buffer without BSA, PGE1, and the platelets were then activated by the addition of thrombin activating peptide-6 (TRAP-6; 25-33 μg/mL, Sigma Aldrich) and incubated for 15 min at 37°C. After activation, the platelets were washed once and resuspended in Tyrode’s buffer. Activated and non-activated platelets were then diluted to a concentration of 200 x 10^9/L with Tyrode’s buffer. In experiments where platelets were incubated with purified C3, the protease inhibitor PMSF (1 mM) was added to the washing buffer to inhibit putative proteases that could cleave C3.

### Flow cytometric analysis of platelet-bound C3

C3 (10-100µg/mL) binding to platelets was detected by FITC-conjugated rabbit pAb anti-C3c antibody (10 µg/mL, DAKO GmbH, Jena, Germany). C3 conformation after binding to the platelets, was monitored using anti-C3 mAbs specific for different neoepitopes in the C3 followed by anti-mouse Ig‐FITC (DakoCytomation). The samples were analyzed using Accuri C6 cytometer (BD) and ∼5000 platelets were analyzed for each sample. Mouse anti-human P-selectin (CD62p, 10 µg/mL, Invitrogen, Thermofisher Scientific, Sweden) was used to confirm platelet activation (22).

To confirm the exposure of phosphatidyl serine on platelets, annexin-FITC was allowed to bind to the platelets (Apoptosis detection kit, BD Pharmingen). In some experiments, platelets were preincubated with Annexin V (25 μg/ml; Sigma-Aldrich) for 30 min at RT prior to addition of native C3 (100 μg/mL) followed by another incubation for 30 mins at RT. Platelets where then washed and incubated with anti-C3c antibodies (see above) and analyzed by flow cytometry.

### Binding of various forms of C3 to platelets and their ability to form AP convertases

Isolated non-activated and TRAP6-activated platelets (100 µL of 50 × 10^9^/L platelets) were incubated with purified C3, C3b, or C3(met) (10 µg/mL) for 45 min at 37°C followed by washing with Tyrode’s buffer, pH 6.5. To evaluate the ability to generate AP convertases, isolated non-activated and TRAP6-activated platelets incubated with C3, C3b or C3(met) (10 µg/mL), as described above, were incubated together with FB (25 µg/mL) both in the absence and presence of properdin (25 µg/mL), at 37°C for 30 min. After washing once with PBS, native C3 (10 µg/mL) and FD (0.5 µg/mL) was added and incubated for 60 min. The reaction was stopped with 10 mM EDTA and the platelets were pelleted and the supernatants analyzed for C3a generation.

### Isolation of PMN and human dermal microvascular endothelial cells and induction of apoptosis

*a. PMN:* Blood was collected in 10 mL sodium heparin (17 IU/mL) tubes (BD Vacutainer) and diluted 1:2 in sterile PBS at RT followed by purification on Ficoll-Paque Plus density gradients (GE Healthcare, Uppsala, Sweden) to isolate PMN. The cells were diluted to 1 × 10^6^/mL in RPMI 1640 medium with L-glutamine (Invitrogen, Paisley, UK) and the cell purity and viability were checked with trypan blue using a Luna II automated cell counter (Logos, Biosystems, Gyeonggi-do, South Korea).

*b. HDMEC:* HDMEC cells (PromoCell GmbH, Heidelberg, Germany) were culture in human endothelial cell medium (ECM [^3^H Biomedical, Uppsala, Sweden]) containing 10% heat-inactivated FCS and the cell purity and viability were checked with trypan blue using a Luna II automated cell counter (Logos, Biosystems, Gyeonggi-do, South Korea).

*c. Apoptosis:* For induction of apoptosis, PMN and HDMEC were incubated in 5 µM camptothecin (Sigma Aldrich) for 4h at 37°C. After wash the HMECs were detached by trypsinization (1mg/mL), suspended at a concentration of 10^6^ cells/mL in ECM.

### Binding of native C3 to apoptotic PMN and HDMEC

The different cell populations were validated by flow cytometry using Annexin-FITC to detect apoptosis (Apoptosis detection kit, BD Pharmingen) according to the instructions of the manufacturer. Non-apoptotic and apoptotic cells were incubated with either purified human C3 (100µg/mL) or human serum-EDTA (10mM) diluted 1:10 corresponding to approximately 100 µg/mL of C3 at RT for 30 min.

After washing, the cells were incubated with C3 (100µg/mL) for 30 min at RT in VBS. Anti-CD16-PerCP from BD (BD Pharmingen) were used for gating of PMN. Anti-C3c-FITC (1/100, Dako) was used for staining for C3, while FITC directly conjugated to Annexin V was used to detect apoptosis (Apoptosis detection kit, BD Pharmingen) according to the instructions of the manufacturer. The cells were fixed with 1% (v/v) PFA-PBS. Using the same detection system, competitive binding of Annexin V and native C3 was tested by sequential addition of the non-labelled Annexin V (Beckman Coulter, Netherlands) or C3 at a concentration of 10-100 µg/mL before adding the opposite labelled protein. The samples were analyzed using CytoFlex S (Beckman Coulter, Uppsala, Sweden).

### Capillary immune electrophoresis and traditional Western blotting

Simple Western™ Wes Capillary immune electrophoresis (CIE) instrument (Bio-Techne, Minneapolis, USA) was used to analyze C3 in the different fractions using one mAb specific for the C3α-chain (C3a; 4SD17.3) and one for the C3β-chain (7D169.1) for detection. C3 bound to platelets isolated from activated and non-activated PRP was also analyzed by CIE with the mAb 7D84.1 against C3α-chain for detection and purified C3 and C3b as controls. C3 bound to non-apoptotic and apoptotic PMNs was analyzed by traditional Western blotting using pAb anti-C3c and 4SD17.3 for detection and C3, C3b and iC3b as controls. For platelets and PMNs, the protease inhibitor PMSF (1 mM) was added from the start to the washing buffers to inhibit putative serine proteases from cleaving C3.

### Immunohistochemistry

Clots were formed in citrate plasma that had been centrifuged once at 3200 x g, leaving only trace amounts of platelets. The supernatant was transferred to new containers where clots were formed by recalcification and addition of thrombin (2 U/mL) followed by incubation at 37°C for 30 min after which the clots were snap frozen, cryosections of 4 µm thickness were prepared and stored at -80°C.

a. Immunofluorescence. Paraformaldehyde 4% fixated blood clots were blocked using 2.5% horse serum for 30 minutes. The clots were subsequently labelled for complement proteins using a primary rabbit polyclonal anti-C3c antibody diluted 1:2000 (Dako, Glostrup, Denmark) and a monoclonal anti-C3a antibody 4SD17.3, diluted 1:200. The clots were incubated overnight in a humid chamber at 4°C. After washing the clots in 10 mM phosphate buffer (Sigma-Aldrich, Saint Louis, USA), pH 7.4, secondary antibodies were applied using the VectaFluor Duet double labelling kit. In brief, cells were clots allowed to interact with a mixture of DyLight 488 anti-rabbit and 594 anti-mouse secondary antibodies (Vector Laboratories Inc., Newark, USA) for 30 minutes. Clots were washed and mounted using 4′,6-diamidino-2-phenylindole (DAPI, Vector Laboratories). Negative controls included an isotype IgG1 (Dako) antibody as well as omission of the primary antibodies.

b. *In situ* proximity ligation assay. For the in-situ proximity ligation assay (PLA, Sigma-Aldrich), the manufacturer’s protocol was followed. In brief, the fixated clots were blocked with a Duolink blocking solution (Sigma-Aldrich) for 60 minutes at 37°C. The clots were then incubated overnight at 4°C with primary antibodies against C3c, diluted 1:2000 (Dako), and C3a, clone 4SD17:3 diluted 1:200. After washing the plus anti-rabbit and minus anti-mouse PLA probes (Sigma-Aldrich) were applied diluted 1:5 in antibody diluent (Sigma-Aldrich) and incubated for one hour at 37°C. Probes were subsequently ligated using Duolink ligation buffer (Sigma-Aldrich), diluted 1:5, and incubated on the clot for 30 minutes at 37°C. The signal from the ligation was amplified by adding in situ detection reagents specific for Texas red. Clots were washed and mounted using in situ mounting media with DAPI (Sigma-Aldrich). To ensure specificity, negative controls were established by omitting the primary antibodies individually, as well as omitting the ligase.

c. Imaging and analysis. Immunofluorescence was analysed using a Nikon Ti2-E AX confocal microscope (Nikon, Tokyo, Japan), equipped with appropriate filters. To obtain optical sections through the clots, images were collected as z-stacks with a step size of 0.5 μm, using the NIS-Elements software (Nikon).

### Liposome preparation

Lipids were dissolved in ethanol, or a chloroform-methanol mixture followed by removal of the organic solvent by evaporation. Liposomes were freshly prepared by hydration of the lipid film in H_2_O (MilliQ grade) followed by hand extrusion 11 times through an 80 nm diameter polycarbonate filter and 11 times through a 30 nm diameter polycarbonate filter at 50-60°C using an Avanti Mini Extruder (Birmingham, Al, USA). The used lipids and the composition of the produced liposomes (% phospholipids) are presented in **Table 1**. The liposomes were characterized concerning both size and zeta potential using a Malvern Zetasizer (Malvern, UK).

**Table 1:** Composition of liposomes presented in **Figure 5**.

Liposome composition	Size (nm)	PDI	Charge (mV)	Appearance panel, color
DPPC: Chol (60:40)	85.1 ± 0.7	0.07 ± 0.02	1.2 ± 0.8	A, Green
DPPC: DPPG (80:20)	N/A	N/A	N/A	A, Magenta
DPPC: Chol : DPPG (40:40:20)	106.4 ± 2.5	0.06 ± 0.04	–51.5 ± 1.1	A, B, E, F, Blue
DPPC: Chol : DPPG (55:40:5)	116.8 ± 0.2	0.06 ± 0.02	–41.1 ± 1.2	B, Light Blue
DPPC: Chol : PS (40:40:20)	94.6 ± 0.6	0.07 ± 0.02	–39.2 ± 1.3	B, C, D, E, F, Red
DPPC: Chol : PS (55:40:5)	97.6 ± 0.4	0.06 ± 0.00	–32.1 ± 0.5	B, Light Red

Used lipids: DPPC: 1,2-dipalmitoyl-sn-glycero-3-phosphocholine (C_40_H_80_NO_8_P) Avanti Polar Lipids (Alabaster, AL, USA); DPPG: 1,2-dipalmitoyl-sn-glycero-3-phospho-(1’-rac-glycerol (C_38_H_74_O_10_PNa): Avanti; Chol: Cholesterol, Sigma–Aldrich (Munich, Germany); PS: 1,2-diacyl-sn-glycero-3-phospho-L-serine, Sigma.

### QCM-D analysis of C3 binding and conformation on liposomes

The quartz crystal microbalance with dissipation monitoring (QCM-D) measurements were performed using a QCM-D Pro from Biolin Scientific AB (Gothenburg, Sweden). A layer of liposomes/bilayer was first allowed to assemble on the surface of the SiO_2_ sensors in the QCM-D instrument by letting the liposome suspension flow over the surface for 30 min. After priming with buffer, C3 (10 μg/mL) in veronal buffered saline (VB^++^; 5 mM barbiturate, pH7.4; 145 mM NaCl; 0.15 mM Ca^2+^; 0.5 mM Mg^2+^) was added for 50 min, followed by mAb 7D84.1 (10 μg/mL) for 20 min, and finally anti-C3c antibody (10 μg/mL) for 20 min.

### Formation of AP convertase and inhibition of FH on liposomes

QCM-D studies were performed only with the liposomes with the composition molar ratio of DPPC: Cholesterol : DPPG (40:40:20). C3 (10 μg/mL) was allowed to bind to the liposome-coated sensors for 50 min, followed by the addition of a mix of FB (10 μg/mL) and properdin (5 μg/mL) for 5 min. Finally, FD (0.5 µg/mL) and additional native C3 (10 µg/mL) were added. In another experiment, SiO_2_ sensors were prepared with liposome/bilayers. C3 was added (10 μg/mL) and allowed to bind for 50 min. FH (5 µg/mL) was added, followed by FB together with properdin.

### Competitive binding of C3 and Annexin V on the liposomes

Four QCM-D sensors were coated with negatively charged cholesterol liposomes, of which two sensors received liposomes with the composition DPPC: Cholesterol : DPPG (40:40:20) and the other two sensors with liposomes of composition DPPC: Cholesterol : PS (40:40:20). Next, Annexin V (0.2µg/mL) was added to two of the sensors, one of each liposome type. Finally, C3 (10 µg/mL) was added to all four sensors.

### Cryo-EM studies of C3 binding and conformation on cholesterol-containing liposomes

Prior to sample application, graphene oxide was applied to all Quantifoil 1.2/1.3–300 mesh grids as previously described (32). Complement C3 (0.2 mg/mL) was incubated with either DPPC: Chol : PS [40:40:20] or DPPC:X:PS [80:20] for 60 mins before grid freezing. An initial blot was performed with 3 μL of C3/liposome solution applied to the graphene oxide-containing grids for 3 minutes. Vitrification was then performed with a Vitrobot Mark IV (Thermo Fisher Scientific) at 25°C and 95% humidity. Grids were blotted in duplicate for 4 seconds and plunged into liquid ethane. Following vitrification, the grids were clipped into autogrid cartridges (Thermo Fisher Scientific) for use with autoloader systems.

All grids were screened and full datasets were collected on a Glacios 200 kV cryo-electron microscope mounted with a Falcon 4i direct electron detector with Selectris energy filter. Data were acquired in EER format at a pixel size of 1.15 Å/pixel (100 kx magnification), 56 e^−^/Å^2^ total electron dose, −0.8 to −2.0 μm defocus range, dose rate of 0.91 electrons pixel^-1^ sec^-1^. Automated collection was performed using the EPU software (Thermo Scientific). A total of 4,600 micrographs were collected and had 2x binning applied and were processed using exposures fractionated into 41 frames for motion correction.

Processing was performed in cryoSPARC v4.6.0 and included initial Patch Motion Correction and Patch CTF being performed before micrograph curation (33). Micrographs were manually curated based on CFT estimations, total frame motion, defocus, ice thickness and contamination. Using the obtained template from the screening dataset, all particle picking was performed with blob picker. The picks totaled ~2 million and were 2x down sampled and extracted with a 400 x 400 box size before being Fourier-cropped to 250 x 250 pixels. Particles were classified into 150 2D classes (10 Å minimum separation distance, 170 - 220 Å circular mask inner-diameter, 60 online-EM iterations, 400 batch size per class). Following 2D class selection, this classification/selection procedure was repeated twice more which resulted in ~896,000 particles suitable for *Ab-initio* reconstruction. *Ab-initio* reconstruction was followed by heterogenous refinement using a few poor density “dummy” classes as seeds for junk particles. Two further rounds of heterogenous refinement seeding resulted in 317,410 particles suitable for masked refinement and reconstruction jobs which produced a density map at ~3.8 Å.

### Statistical analysis

All experiments were repeated at least three times. Data are presented as mean ± SEM or as representative images. Data were analyzed using paired t-tests, if not more than two variables were analyzed when one-way ANOVA was applied followed by multiple comparison. Spearman correlation test was performed for the linear regression analysis. All data were analyzed using GraphPad Prism 10 for Mac OS version 10.4.0 (GraphPad Software, La Jolla, CA, USA). The calculated P-values were defined as follows: *p < 0.05, **p < 0.001, ***p < 0.001, and ****p < 0.0001.

## Results

### Identification of various conformationally changed forms of soluble C3(H_2_O)

**Figure 1:**
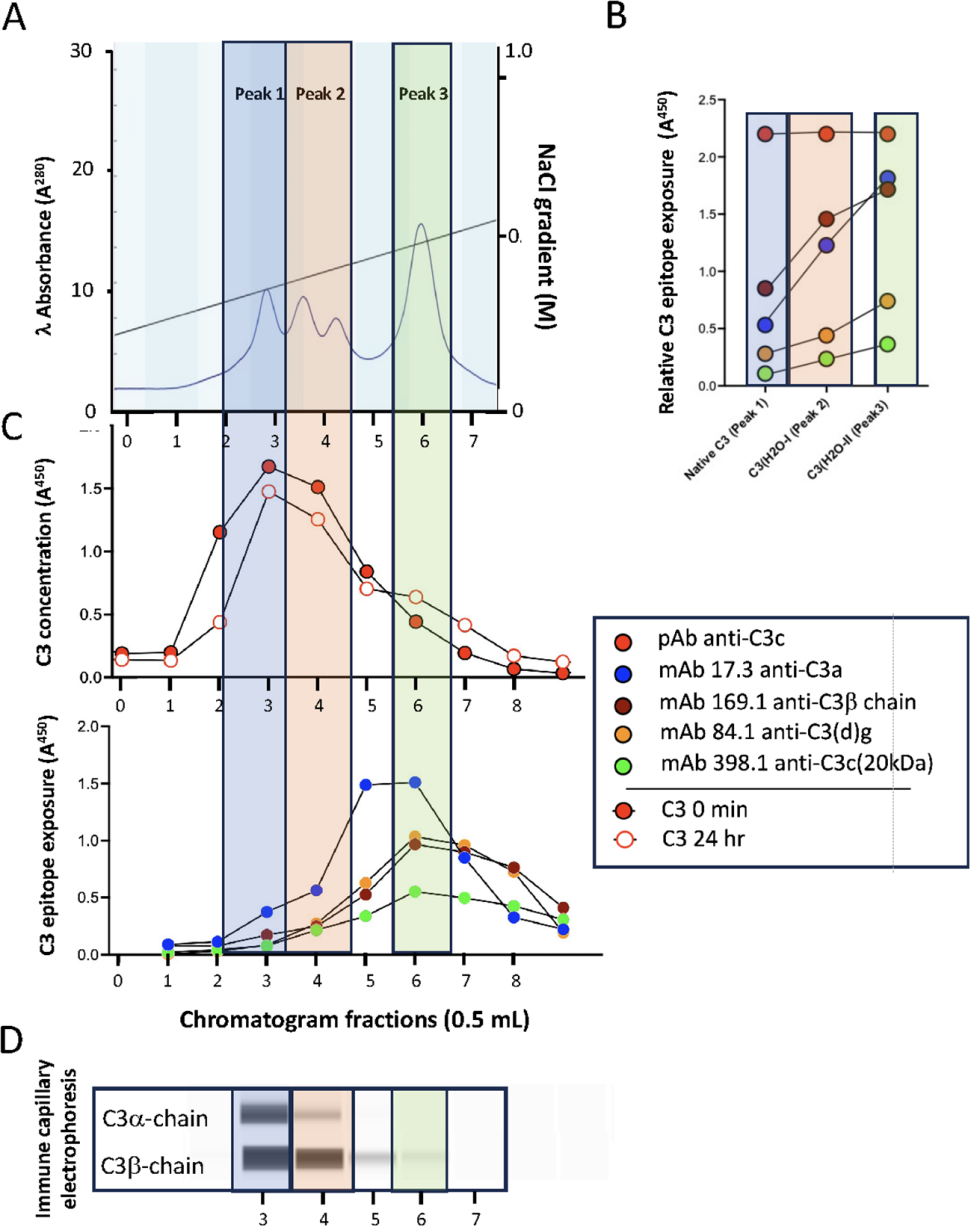
Separation and characterization of C3(H_2_O). **(A)** To characterize C3(H_2_O), native C3 was exposed to 7 freeze/thaw cycles at -20°C for 30 min followed by 15 mins at RT and separated by Mono S Cation exchange chromatography. We have previously identified native C3 (peak 1), an intermediate form of C3(H_2_O) (peak 2; 1–2 peaks) and finally, what is considered to be C3(H_2_O) (peak 3). By following the absorbance at 280nm, we confirmed this pattern. The peaks were indicated with blue (peak 1), beige (peak 2) and green (peak 3). **(B)** The conformation of C3 was followed by sandwich ELISA using capture mAbs against conformational neoepitopes on C3. The same equimolar concentrations of C3 were used for all peaks, which was confirmed by the quantitative C3 ELISA (red). The C3a neoepitope detected by mAb 4SD17.3 (blue), demonstrated a continuously stronger exposure of the epitope from native C3 (Peak 1) to C3(H_2_O) of Peak 3. Also, mAbs against other conformational epitopes (clones 7D169.1 [brown], 7D84.1 [orange], 7D398.1 [green]) were used. **(C)** One mL of freshly prepared human serum was separated by the same Mono S Cation exchange chromatography as for purified C3. Two serum preparations were run: the initial serum immediately after preparation and the second after a 24 h incubation at 37°C. The fractions of the chromatogram were analyzed for C3 by quantitative sandwich ELISA (upper) and the serum incubated at 37°C for 24h by the same conformational epitope ELISAs (lower) as in panel B (with the same color code). Most of the conformationally changed C3 was found between peaks 2 and 3. **(D)** The fractions of the serum incubated at 37°C for 24h were analyzed by CIE using one mAb specific for the C3α-chain (C3a; 4SD17.3) and one for the C3β-chain (7D169.1).

In previous studies, we and others have identified 3–4 major peaks after separation of purified C3(H_2_O) by cation exchange chromatography (9), corresponding to native C3 (peak 1), to an intermediate form of C3(H_2_O) (peak 2) and finally to C3(H_2_O) (peak 3) (**Figure 1A**). Here, this conversion was monitored by sandwich ELISAs using mAbs against conformational neoepitopes of C3 (**Figure 1B**). It demonstrated a continuous increase in the exposure of the C3a neoepitope detected by mAb 4SD17.3, from a weak exposure in the native C3 in Peak 1, to a strong exposure in C3(H_2_O) of Peak 3, confirming a gradual conformational change in the protein (**Figure 1B**). The presence of the C3a epitope confirmed the presence of C3a in the C3 molecules and thus the absence of cleavage at the convertase cleavage site into C3a and C3b. The fractions were also tested with three other mAbs against conformational epitopes (clones 7D169.1, 7D84.1, 7D398.1), which all confirmed the conformational change in the molecule.

### Identification of conformationally changed forms of soluble C3(H_2_O) in serum

C3 in freshly prepared serum and in serum incubated for 24 h at 37°C was separated by the same method. These chromatograms did not explicitly show individual C3 peaks due to the abundance of other plasma proteins present (not shown). However, using the quantitative sandwich ELISA employing pAb anti-C3c, two distinct peaks of C3 were identified (**Figure 1C**; upper panel). The first major peak (peak 1) corresponded to native C3. The other peak in the position of peak 3, was displayed as a knee in the chromatogram of the 24 hr incubated serum. Using the sandwich-ELISAs, this peak exposed the neoepitops including the one in C3a (**Figure 1C**; lower panel). Capillary immune electrophoresis (CIE) analyses (**Figure 1D**), employing mAb anti-C3β-chain (7D169.1) was able to detect C3β chain present in fractions 3-6, in proportion to the total amount of C3 in these fractions (**Figure 1C** upper panel). Intact C3α-chain (mAb 4SD17.3) was only present in peak 1 (fraction 3) and hardly visible in peak 2 (fraction 4). The intact C3α-chain confirms the presence of native C3 in peak 1 since factor I in serum is not able to cleave native C3. By contrast, C3(H_2_O) is susceptible to factor I cleavage, suggesting that generated C3(H_2_O) is immediately inactivated into iC3H_2_O (cf iC3b) in serum (34).

### Conformationally changed native C3 binds to activated platelets

**Figure 2:**
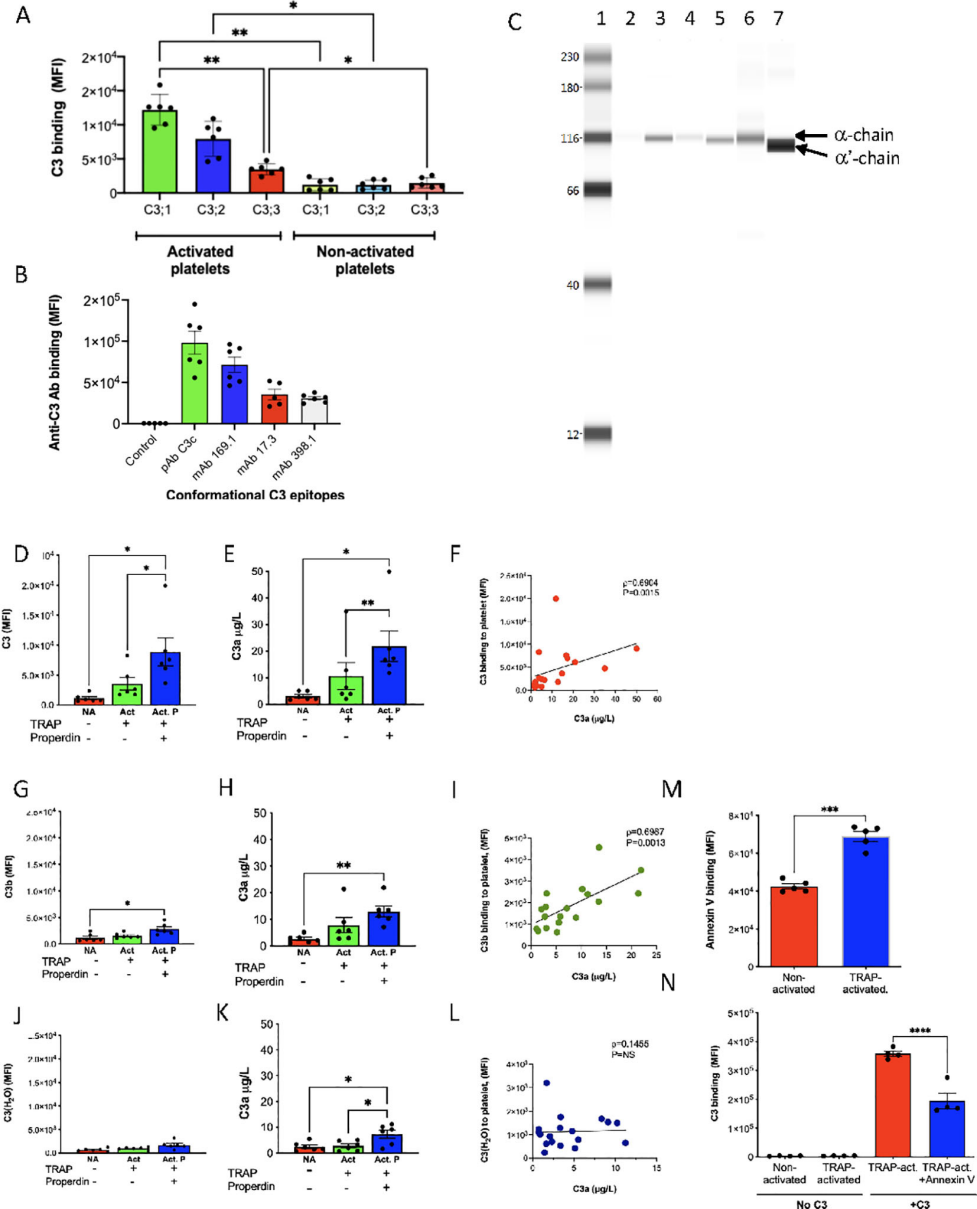
Binding of different forms of C3 to platelets. **(A)** To investigate if it is preferably native C3 or any of the C3(H_2_O) forms that bind to activated platelets, individual fractions of purified freeze-thawed C3(H_2_O) isolated by Mono S cation exchange chromatography (see **Figure 1A**), were added at the same concentration (10µg/mL), to both activated and non-activated platelets. The C3 binding was detected by flow cytometry using pAb anti-C3c antibody (mean ± SEM; n=6). **(B)** Native C3 (10µg/mL) was allowed to bind to activated platelets. The conformation of bound C3 was characterized by flow cytometry using mAbs directed to neoepitopes including in C3β chain (mAb 7D169.1), C3a (mAb 4SD17.3), and C3c 20kDa α-chain polypeptide (mAb 7D398.1) (n=6). **(C)** To determine the polypeptide structure of C3 bound to platelets during activation, we analyzed the platelets by CIE with purified C3 and C3b as controls. Lane 1, biotinylated ladder 12–250 kDa; lane 2, non-activated PRP donor 1; lane 3, TRAP6-activated PRP donor 1; lane 4, non-activated PRP donor 2; lane 5, TRAP6-activated PRP donor 2; lane 6, native C3 (10 µg/mL); and lane 7, C3b (10 µg/mL). Arrows indicate C3 α-chain and C3b α´-chain. The non-activated platelets bound only trace amounts of C3 while activated platelets bound substantially more native C3. Only C3α-chain of C3 but no C3bα´-chain was found in lanes 2-5. **(D–L)** To test the ability of platelet-bound C3 to form AP convertases, we allowed 10 µg/mL of native C3 **(D)**, C3b **(G)**, and C3(met) **(J)** to bind to isolated purified platelets in the absence and presence of 33.5 µM TRAP6 (n=6). The C3 binding were quantified by flow cytometry. It was found that native C3 was the superior binder to the activated platelet surface, while C3b bound substantially less to the platelets. C3(met) was the poorest binder to the platelets. The binding of all forms of C3 was enhanced in the presence of properdin. Platelet bound native C3, C3b, and C3(met) as in **(D, G, J)** were also incubated with FB, FD and C3 to create an AP C3 convertase. The formed convertases were functionally active as demonstrated by the generation of C3a **(E, H, K)**. The C3a generation was also amplified by properdin. The amount of platelet bound C3, C3b, and C3(met) and the generation of C3a were correlated in **(F, I, L)** (**(D-L)** n=6 in all experiments). **(M, N)** To investigate whether activated platelets were exposing phosphatidyl serine, binding of Annexin V-FITC was assessed **(M)**. Unlabeled annexin V (25µg/mL) was also used to show that Annexin V was competing for the same binding site on platelets as C3 (100µg/mL) **(N)** (n=6). Data presented as mean ± SEM, *p < 0.05, **p < 0.001, ***p < 0.001, ****p < 0.0001. One-way ANOVA followed by multiple comparison **(A, B, D, E, G, H, J, K)**: Spearman rank correlation **(F, I, L)**; paired t-test **(M, N)**.

We have previously shown that intact C3 binds to activated platelets in PRP anticoagulated with EDTA or lepirudin and to isolated activated platelets (22). The tentative conclusion was that the C3 was in its C3(H_2_O) form. To investigate whether it is preferably native C3 or any of the C3(H_2_O) forms that bind to activated platelets, individual fractions of purified freeze-thawed C3(H_2_O) isolated by Mono S cation exchange chromatography (see **Figure 1A**), were added, at the same concentration, to both activated and non-activated platelets (**Figure 2A**). Flow cytometry analysis shows that it is mainly native C3 (peak 1) that binds to activated platelets. C3(H_2_O) intermediate (peak 2) also binds to some extent, while only very small amounts of C3(H_2_O) (peak 3) could be detected. No C3, regardless of conformation, binds to non-activated platelets.

The conformation of C3 after binding to activated platelets, was characterized using the mAbs directed to neoepitopes described above (**Figure 2B**). It turned out that several of the C3 epitopes were exposed after binding to platelets, including in the C3β chain (mAb 7D169.1), C3a (mAb 4SD17.3), and C3c 20kDa α-chain polypeptide (mAb 7D398.1).

CIE was used to analyze C3 bound to platelets isolated from activated and non-activated PRP (**Figure 2C**). As expected, the non-activated platelets bound only trace amounts of native C3 and no C3b. The activated platelets bound C3 with an intact α-chain without any traces of α’-chain (C3b). This band was visualized with mAb 7D84.1 to C3d,g, which can detect both the α-chain and the α’-chain of C3 and C3b, respectively, ruling out the presence of C3b on the platelet surface.

### Formation of AP C3 convertases with platelet-bound C3

Native C3, C3b, and C3(H_2_O) (C3[met]) were allowed to bind to isolated non-activated and TRAP6-activated platelets in the presence and absence of properdin. The platelets were then analyzed by flow cytometry using pAb anti-C3c which is able to detect intact C3, C3(H_2_O), and C3b. Again, C3 bound strongly to the activated platelets while non-activated platelets did not bind any form of C3. Interestingly, the binding was much enhanced by properdin (**Figure 2D**). Both C3b (**Figure 2G**) and C3(met) (**Figure 2J**) were poor binders, although their binding was slightly increased in the presence of properdin. The platelet with bound C3, C3b, and C3(met) were incubated with FB and followed by the addition FD and native substrate C3 to the formed AP convertases. The level of convertase formation was then evaluated by measuring the amount of generated C3a. The bound native C3 generated most C3a, followed by C3b and C3(met) (**Figures 2E, H, K**). As expected, convertase formation increased in the presence of properdin. When the amount of C3a was correlated with the amount of bound C3 on the platelet surface (non-activated and activated ± properdin), strong correlations were obtained for bound native C3 and C3b, supporting that native C3 transforms into a C3b-like form of C3 upon binding to the platelet surface (**Figures 2F, I**). Also, on a molar basis native C3 seemed to be equally efficient as C3b. No correlation was found between C3a generation and platelet-bound C3(met) (L), implying that C3(met) is an inefficient convertase companion.

The phospholipid-scrambled membranes of the activated platelets was shown as binding of exogenously added Annexin V (**Figure 2M**). Annexin V was able to partially inhibit the binding of C3 to activated platelets (**Figure 2N**).

### C3 in TRAP6-activated blood during clotting and platelet aggregation

**Figure 3:**
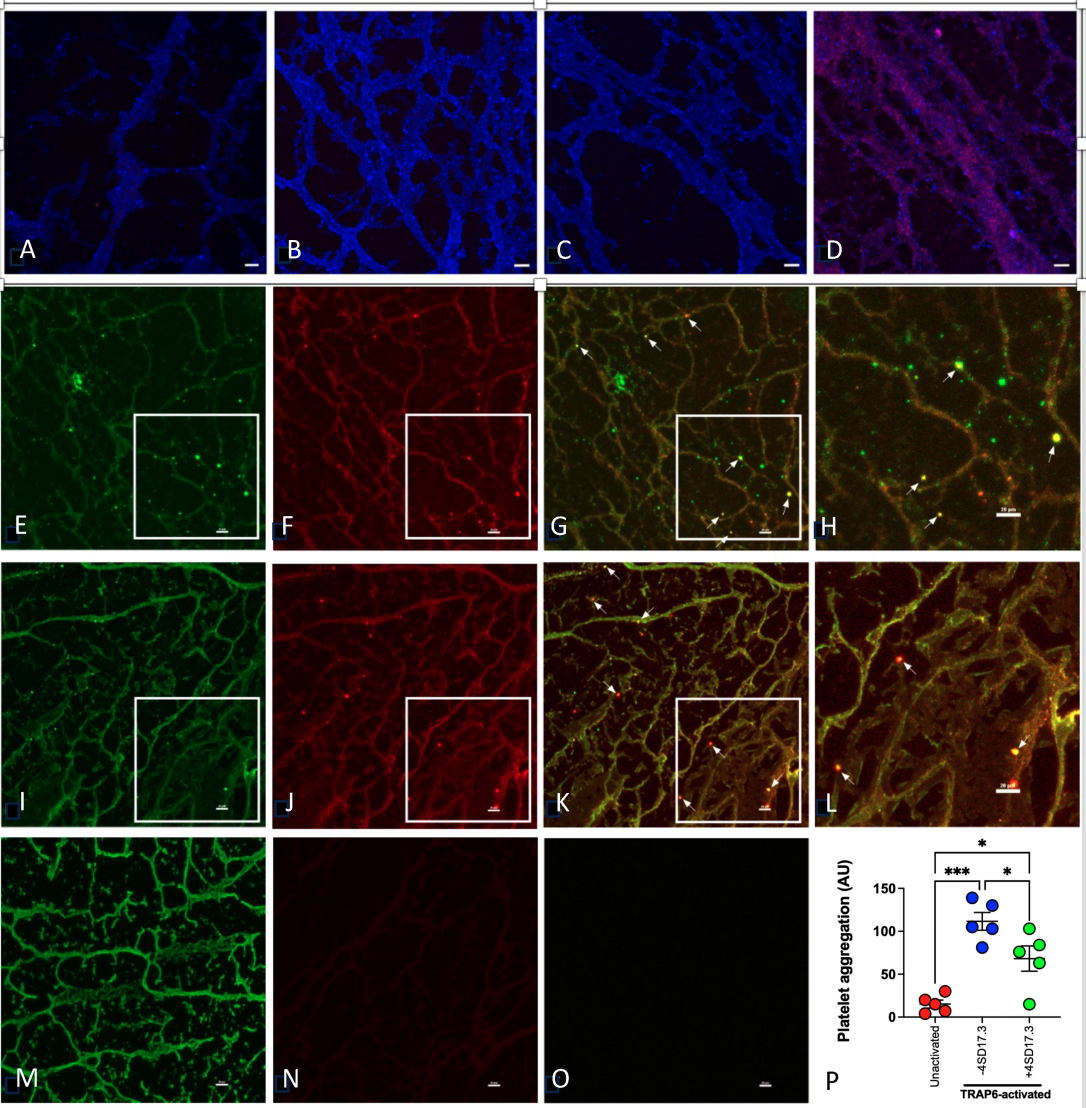
C3 bound to *in vitro* formed clots. Clots were formed in plasma that had been centrifuged only once at 3200g, leaving trace amounts of platelets left in the supernatant. The sections were stained using a proximity ligation assay in panels **(A-D)**. **(A)** control (both antibodies, without ligase), **(B)** pAb anti-C3c, **(C)** mAb anti-C3a (4SD17.3), and **(D)** both anti-C3a and anti-C3c resulting in hybridization enhanced by Texas red (purple color). Fluorescence staining were used in panels **(E)** P-selectin (CD62P), **(F)** C3a, **(G)** an overlay of **(E)** and **(F)** and **(H)** a magnification of **(G)** Panels **(I)** C3c, **(J)** C3a, **(K)** an overlay of **(I)** and **(J)**, and **(L)**. a magnification of **(K, M)**. fibrinogen, **(N)** non-immune mouse Ig and **(O)** non-immune rabbit Ig. Scale bar: 20 µm. P: Platelet aggregation in TRAP6-activated citrated normal human blood was assessed. The samples were treated with or without 10µg/mL mAb 4SD17.3 for 15 min at RT before the samples were tested in the device. A significant inhibition of aggregation was observed in the mAb anti-C3a treated samples (n=5). One-way ANOVA followed by multiple comparison **(L)**. Data presented as mean ± SEM, *p < 0.05, ***p < 0.001.

To investigate whether intact C3 could be detected in *in vitro* formed blood clots, blood clot sections were firstly stained using proximity ligation. **Figures 3A-C** shows negative controls A: both antibodies no ligase, B: pAb anti-C3c alone; C: mAb anti-C3a (4SD17.3) alone. In Panel D hybridization enhanced by Texas red (purple color) shows that the C3a epitope and the rest of C3 (C3c) are located together on platelets and platelet microparticles. This was confirmed using traditional immunofluorescence staining for fibrinogen, P-selectin (CD62P), C3 (pAb anti-C3c) and C3a (mAb 4SD17.3). **Figures 3E-H** show that CD62 positive platelets also bind the mAb anti-C3a. In the next panels (**Figures 3I–O**) it is demonstrated that C3a positive platelets are also positive for C3c, corroborating the presence of intact C3 on the platelets. By using a multiplate device, the ability of intact C3 to inhibit aggregation in whole blood was tested. In **Figure 3P** it is shown that mAb 4SD17.3 against a neoepitope in C3a partially inhibits the aggregation. This supports that intact platelet-bound C3(H_2_O) was targeted, previously shown to mediate platelet/leukocyte complex formation (26).

### Binding of C3 to apoptotic PMNs and HDMEC

**Figure 4:**
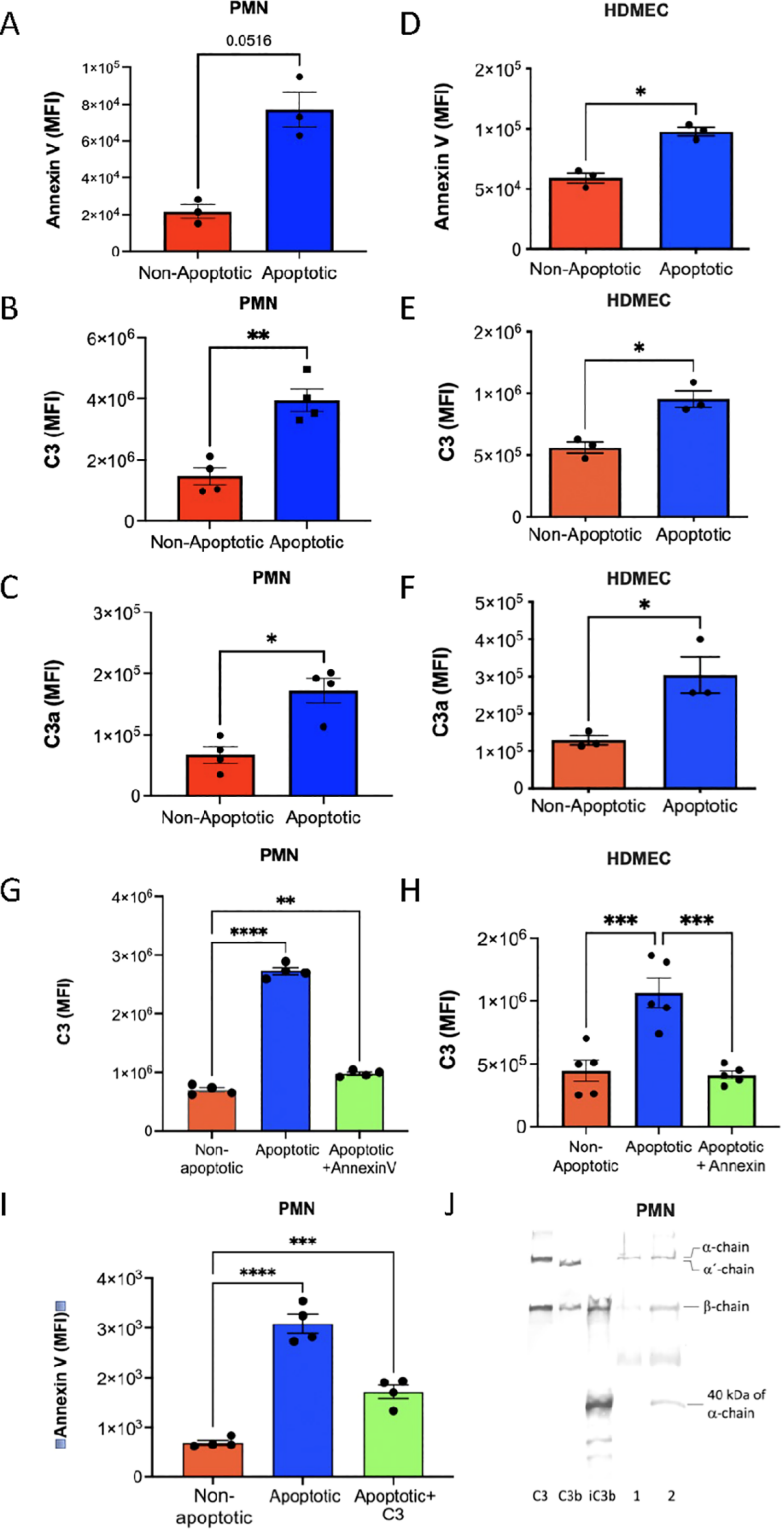
Binding of native C3 to apoptotic PMN and HDMEC. PMN and HDMEC were treated with camptothecin (5 µM). For flow cytometry, anti-CD16 was used to identify the PMN. Native C3 (100 µg/mL) was added to the PMN and the HDMEC, followed by incubation for 30 min at RT in the presence of PMSF (1 mM). The samples were analyzed by flow cytometry. Panel A shows binding of Annexin V, B binding of C3 detected by anti-C3c and C binding of mAb 4SD17.3 anti-C3a to PMN, (n=4). **(D–F)** the corresponding binding of C3 in serum-EDTA (10 mM) to HDMEC (n=3). Competitive binding of Annexin V and native C3 was tested by adding non-labelled Annexin V before testing C3 binding to PMN (**(G)** (n=4)) and to HDMEC [**(H)** (n=5)]. In panel I the reciprocal binding of annexin to PMN is shown (n=4). Paired t-test **(A-F)**; one-way ANOVA followed by multiple comparison **(G-I)**. Data presented as mean ± SEM, *p<0.05, **p < 0.001, ***p < 0.001. **(J)** Western blot of the binding of native C3 to PMN before and after induction of apoptosis. Controls: purified C3, C3b and iC3b, lane 1, C3 binding to non-apoptotic PMNs and lane 2 the corresponding binding to apoptotic PMNs. Immunoprecipitation of C3 was avoided to prevent unspecific proteolytic cleavage. Controls and lanes 1 and 2 are detected with pAb anti-C3c.

Apoptosis was induced in PMN and HDMEC by camptothecin. Annexin V indicated that the cells were apoptotic (**Figures 4A, D**). Native C3 was added to both the non-apoptotic and the apoptotic cell populations, and C3 binding was assessed by flow cytometry using pAb anti-C3c (**Figures 4B, E**). It clearly showed that the apoptotic cells bound much more C3 compared to the normal (non-apoptotic) cells. The same analysis was done with mAb 4SD17.3 which showed that the C3a domain of bound C3 was exposed in a similar way as on activated platelets (**Figures 4C, F**).

Annexin V binds negatively charged phosphatidyl serine exposed on the apoptotic cell membrane. When Annexin V was allowed to bind to the apoptotic PMN and HDMEC before C3, the C3 binding was almost completely inhibited (**Figures 4G, H**). Similarly, Annexin V binding to PMN was largely reduced if C3 was added before the addition of Annexin V (**Figure 4I**). This suggests that C3 binds to the same sites as Annexin V on the apoptotic cell membranes.

Western blotting using the pAb anti-C3c showed that C3 bound to the apoptotic cells was non-cleaved, i.e., the detected C3 had an intact α-chain (**Figure 4J**).

### Binding of C3 to liposomes of various composition

**Figure 5:**
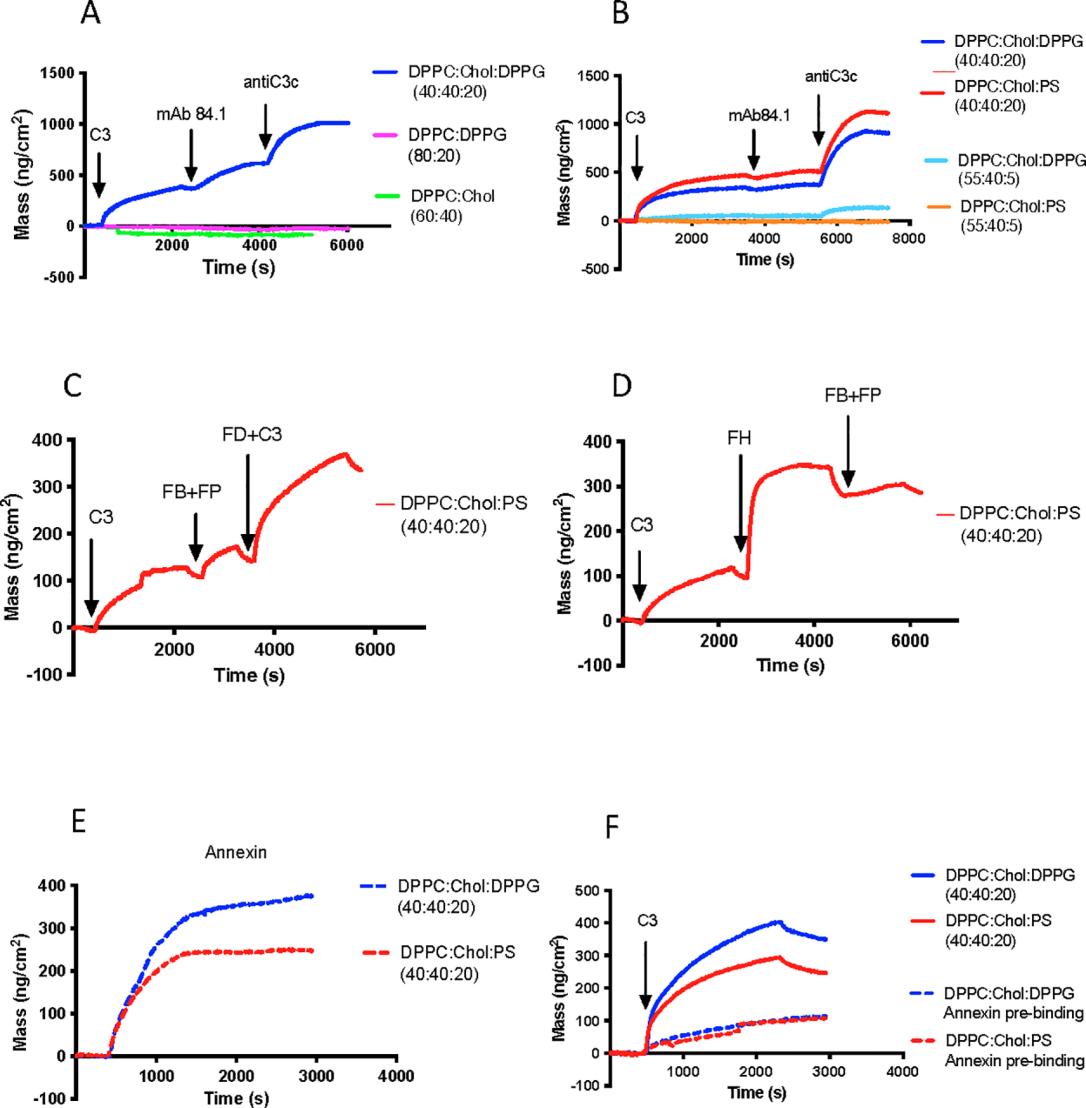
C3 binding, conformation and function on liposomes QCM-D analysis which allows real time analysis of C3 binding and conformation on different types of liposomes with various compositions, prepared and analyzed as described in **Table 1**. In all panels, a layer of liposomes/bilayer was first allowed to assemble on the surface of the SiO_2_ sensors in the QCM-D instrument by flowing the liposome suspension over the surface for 30 min. **(A, B)** After priming with buffer, C3 (10 μg/mL) in VB^++^-buffer was added for 50 min., followed by mAb 7D84.1 (10 μg/mL) for 20 min, and finally anti-C3c antibody (10 μg/mL) for 20 min. Liposomes in **(A)** DPPC: Chol : DPPG (40:40:20), blue, (negative with cholesterol); DPPC: DPPG (80:20), magenta, (negative without cholesterol); DPPC: Chol (60:40), green, neutral with cholesterol). Liposomes in **(B)** Negative net charge with 20 mol% neg charged (DPPC: Chol : PS [40:40:20]; red) (DPPC: Chol : DPPG [40:40:20]; blue) and 5 mol% negative charged phospholipids (DPPC: Chol : PS [55:40:5]; light blue) (DPPC: Chol : DPPG [55:40:5]; light red). Only the liposomes with negative net charge with cholesterol and 20 mol% neg charged phospholipids bound anti-C3c antibodies. The binding of mAb 7D84.1 indicated that the C3 molecule had undergone a conformational change. **(C)** Since C3 only bound to the negatively charged liposome surface containing cholesterol, the subsequent QCM-D studies were performed only with the liposomes with the composition molar ratio of DPPC: Cholesterol : PS (40:40:20). Formation of AP convertases was achieved by letting C3 (10 μg/mL) bind to the liposome-coated sensors for 50 min, followed by the addition of a mixture of FB (10 μg/mL) plus properdin (5 μg/mL) for 5 min. Thereafter, FD together with more C3 was added. **(D)** SiO_2_ sensors were prepared with liposome/bilayers. C3 was added and allowed to bind for 50 min. Next, factor FH (5 µg/mL) was added, followed by FB and properdin. Finally, FD together with more C3 was added. The addition of FH inhibited the generation and deposition of C3b to the surface. **(E)** Annexin V was first allowed to bind to two parallel sensors coated with liposomes/bilayers either with the composition DPPC: Chol : DPPG [40:40:20] (blue dotted line) or DPPGCChol: PS [40:40:20] (red dotted line). **(F)** C3 was then added to the two liposome/bilayer surfaces that had bound Annexin V (blue dotted line and red dotted line), and two sensors only coated with DPPC: Chol : DPPG (blue line) and DPPC: Chol : PS (red line) liposomes.

When the liposomes were tested by Quartz Crystal Microbalance with Dissipation (QCM-D), it was found that C3 were deposited on the liposome surfaces containing 20 mol% negatively charged phospholipids and cholesterol, i.e. (DPPC: Chol : DPPG; 40:40:20) and (DPPC: Chol : PS; 40:40:20). Liposomes composed of only net neutral phospholipids and cholesterol (DPPC: Chol; 60:40) and negatively charged liposomes without cholesterol (DPPC: DPPG; 80:20) did not bind any C3 at all (**Figure 5A**).

The conformation of C3 bound to the DPPC: Chol : DPPG and to DPPC: Chol : PS liposomes, was evaluated using neoepitope mAb anti-C3 7D84.1, indicating that the liposome binding had induced a conformational change of the C3 molecule resembling that of surface-bound C3b/iC3b (30). The amount of C3, independent of conformation, was confirmed by a pAb anti-C3c antibody (**Figure 5B**). Interestingly, liposomes with only 5 mol% negatively charged phospholipids and cholesterol, did not bind any C3 either. Thus, 20 mol% of negatively charged phospholipids along with cholesterol were required for C3 binding to the liposome surface. Whether the negatively charged phospholipids were of the PS or DPPG type, did not affect the outcome (**Figure 5B**). Both liposomes bound approximately the same amount of C3. The properties and composition of the liposomes that bound native C3 resemble those of the membranes of activated platelets.

### Formation of AP C3 convertases on negatively charged liposomes containing cholesterol

The negatively charged and high cholesterol-containing liposome surface (DPPC: Chol : PS [40:40:20]) was also tested for its ability to support AP convertase formation. After binding of native C3, the surface was sequentially exposed to FB and properdin followed by FD and additional C3. Both liposomes were able to form AP C3 convertases, which were downregulated by FH. The AP convertase formation with DPPC: Chol : PS [40:40:20] is shown in **Figure 5C** and the corresponding inhibition with FH in **Figure 5D**.

### Competitive binding of C3 and Annexin V on the liposomes

To find out if C3 and Annexin V compete for the same type of binding sites also on the liposomes, Annexin V was first allowed to bind to negatively charged cholesterol-containing liposomes with the composition DPPC: Cholesterol : DPPG (40:40:20) and DPPC: Cholesterol : PS (40:40:20), which showed a high degree of binding (**Figure 5E**). When C3 was added to the Annexin V pre-coated liposome surfaces a significantly smaller mass of C3 was bound, compared to C3 allowed to bind directly to the liposomes (**Figure 5F**). This demonstrates that Annexin V and C3 compete for the same binding sites on the cholesterol-containing negatively charged liposome surfaces.

### Conformationally changed native C3 selectively binds to negatively charged liposomes containing cholesterol

**Figure 6:**
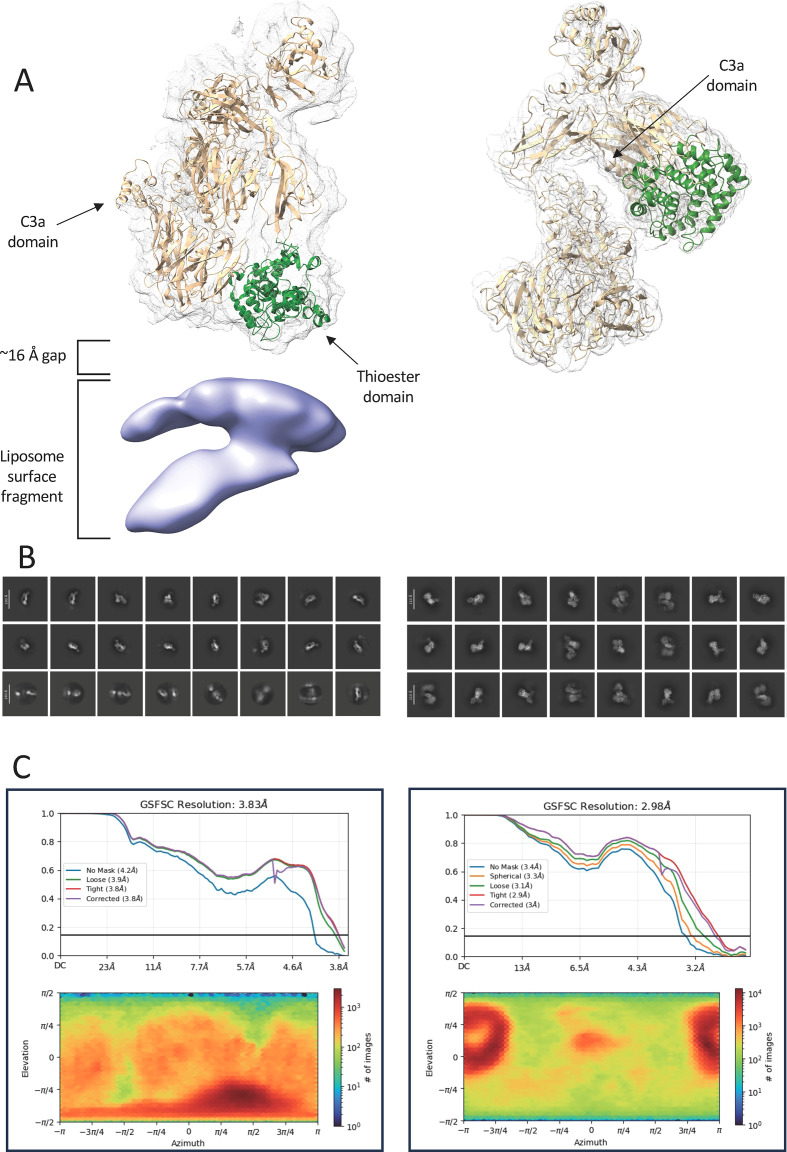
C3 conformations after contact with negatively charge liposomes with and without cholesterol **(A)** The cryo-EM electron densities of the C3b-like (left) and inactive (right) conformations. Density attributed to the C3a domain is present in both conformations. The thioester domain has been highlighted (green) for comparison and exhibits a significant reorientation in the presence of the liposomal surface. **(B)** 2D classification examples of aligned C3b-like particles (left) and inactive C3 (right) from cholesterol-containing and cholesterol-free environments, respectively. **(C)** GSFSC curves and particle orientation distribution heatmaps for both C3b-like particles (left) and inactive C3 (right).

The surface binding of conformationally altered C3 to negatively charged and high cholesterol-containing liposomes was investigated using structural techniques. Accordingly, cryo-EM was employed to uncover the structural basis of binding and the species responsible for surface interaction. Using the lipid composition of DPPC: Chol : PS [40:40:20] and incubating the extruded liposomes with native C3 in a 1:30 ratio, we found that native, conformationally changed C3 was closely associated with the liposomal surface (**Figure 6A**, left panel). The electron densities for all domains were accounted for as well as a significant translocation of the anaphylatoxin C3a domain. Importantly, the thioester was the closest domain to the liposomal surface, positioned approximately 16 Å adjacent. Repeating the microscopy experiment using cholesterol absent liposomes (DPPC: PS [80:20]) maintained the native C3 conformation (35) (**Figure 6A**, right panel). Additionally, no protein was able to be observed near the surface of liposomes, suggesting that the presence of cholesterol is a key feature which drives this protein-surface interaction. 2D classification examples of aligned C3(H_2_O)-like C3 (**Figure 6**, left panel) and inactive C3 (**Figure 6**, right panel) from cholesterol-containing and cholesterol-free environments, respectively, are presented and GSFSC curves and orientation distribution heatmaps for both are presented in **Figure 6C**.

## Discussion

Here, we describe an activation mechanism of native C3 that admits direct recognition of altered-self structures and that takes place on cholesterol and phosphatidyl serine scrambled cell membranes. It allows a non-proteolytic activation of C3 that focuses AP activation to specific target surfaces, distinctive of the present concept of a fluid-phase initiating AP convertase. It is also a shortcut to opsonization of scrambled cell membranes without release of inflammatory peptides, facilitating binding to the phagocytic complement receptors CD35 and CD11b/CD18 (26).

Soluble C3(H_2_O) is the proposed initiator of the AP. Separation of *in vitro*-produced C3(H_2_O) by ion exchange chromatography, yields 3–4 populations of which the first one is remaining native C3 (9, 36). To confirm that the same molecular forms of C3 exist in blood, we separated human blood-serum using the same technique. It confirmed the presence of C3(H_2_O), but only after incubation for 24h at 37°C. However, the α-chain of these forms of C3 were cleaved into iC3(H_2_O), most likely by factor I (9). iC3(H_2_O) (cf iC3b) is unable to form an AP convertase which makes soluble C3(H_2_O) not the first choice as initiator of the AP but can definitely be taken up by cells by binding to C3 receptors such as CD11b/CD18 (10, 37).

The finding that C3(H_2_O) is found on activated platelet surfaces has been interpreted to be the binding of already formed C3(H_2_O) from the fluid phase (25). But this explanation does not fit with the findings that C3(H_2_O) is immediately cleaved to iC3(H_2_O) and that activated platelets exposed to purified native C3 and to C3 in EDTA plasma bind C3 and not C3(H_2_O) to the surface. Now, when we investigated the binding in detail, we found, unexpectedly, that native C3 had the highest affinity for activated platelets in comparison to C3b and C3(H_2_O) (both C3(met) and C3(FT)). We also demonstrated that properdin was an efficient cofactor for the binding of native C3 to the surface and for the formation of AP convertases on TRAP6-activated platelets (21, 25).

Upon activation of platelets by TRAP6, platelets undergo a flip/flop reaction in the membrane where phosphatidyl serine is transferred from the inner leaflet of the membrane to the outer, exposing negative charge (38). Annexin V is a specific marker of this flip/flop reaction (39, 40). When Annexin V is allowed to bind to the activated platelets before C3, the binding of C3 was totally blocked. Under reciprocal conditions Annexin V is inhibited. This supports that C3 has affinity for scrambled membranes exposing phosphatidyl serine (38).

A conformational change was found upon binding of native C3 to activated platelets. The conformational change was associated with exposure of the conformational neoepitopes of the C3(D) epitope cluster, similar to those in fluid-phase C3(H_2_O) (19, 29). Corroborating that the thioester is broken in the platelet-bound C3 is that exposure of these epitopes is closely linked to breakage of the thiol ester and gross changes of the conformation (41).

The platelet-bound C3 was able to form an AP convertase as shown by the generation of C3a in the presence of FB, properdin and later added FD and C3. The initially bound activated native C3 generated much more C3a than both adsorbed C3b and particularly C3(H_2_O) (i.e. C3(met)/C3(FT)). This reveals that native C3 is bound and activated into C3(H_2_O) on the platelet surface in a configuration that is different compared to C3(H_2_O) directly adsorbed to the platelet surface. Other functional effects that can be attributed to surface-bound C3(H_2_O) have previously been reported. We have shown that platelet-bound C3(H_2_O) acts as ligand for both CD35 (CR1) and CD11b/CD18 (CR3) enabling complex formation between platelets and leukocytes (both PMNs and monocytes) (22, 26). This interaction was corroborated in the present study in multiplate aggregation experiments where the mAb anti-C3a antibody inhibited platelet aggregation by approximately 50%. This is similar to the effect previously reported to occur using multiplate analyses when granulocytes were depleted from whole blood (42).

The findings in the platelet experiments encouraged us to also investigate apoptotic cells since a similar membrane scrambling mechanism exposing phosphatidyl serine, occurs in cell membranes during apoptosis (43). Apoptosis was induced in PMN and HDMEC and the apoptotic cells were shown to bind native C3. The binding of C3 to the apoptotic cells was inhibited by preincubating the cells with Annexin V, corroborating that the flip/flop mechanism exposing phosphatidyl serine on the cell surface is involved in the binding of C3 to the membrane. We and others have previously shown that C3 binds to liposomes (24). In the present study, by using phospholipid-containing liposomes, we showed that the binding of native C3 was most efficient to negatively charged (DPPG or phosphatidyl serine) particles containing cholesterol, while negatively charged liposomes without cholesterol did not bind C3. The negatively charged/cholesterol membranes resemble those of activated platelets and apoptotic cells, supporting that those lipids of the membranes are responsible for the binding of native C3 (43). Also, only C3 bound the negative phospholipid/cholesterol-containing liposomes were able to form an AP convertase. In addition, the C3 binding was inhibited by Annexin V, demonstrating resemblance with platelet- and apoptotic cell-bound C3.

The precise molecular mechanism is not clear. Proteolytic cleavage of C3 into C3a and C3b has been ruled out as mechanism activation, both in previous and in the present study by flow cytometry, ELISA or by SDS-PAGE/CIE analyses demonstrating intact C3α-chain. Moreover, bound native C3 did not form covalent bonds with large molecular cell membrane components, since no C3α-chain in complex with other molecules (resulting in higher molecular weight species) was detected.

The fact that phospholipid membranes with a negative charge were able to activate native C3, suggests that the initial contact may be facilitated by charge, since that the isoelectric point of C3 is pH 5.7 (44) making C3 negatively charged at pH 7.4, but there are several positively charged clusters in the molecule, e.g., the anaphylatoxin region (C3a) (35). The liposome-bound C3 exposed the epitopes of 7D398.1 and 7D169.1, demonstrating that C3 had undergone a conformational change. The requirement of cholesterol in the membrane to activate C3 may be due to an interaction between the hydrophobic cholesterol and the hydrophobic pocket that contains and protects the concealed thioester in C3. Tentatively, this interaction would make the thiol ester accessible to nucleophilic attack by nucleophilic groups (35).

To investigate this, the structure was further investigated by the cryo-electron microscopy that supported our findings that C3 is indeed activated by liposomes containing cholesterol and phosphatidyl serine. A significant conformational change was observed, with the protein shifting from the initial 120 Å native form to a 160 Å activated C3(H_2_O)-like conformation. As others have observed (45), this change is facilitated by the release of the MG3 domain and translocation of the C3a domain (35). The conformationally altered C3 shows a head-on interaction between the thioester domain and the surface of the DPPC: Chol : PS phospholipid. The 16 Å distance separating the two species is likely facilitated by the presence of at least a single cholesterol as each molecule is of a comparable length. While the resolution was too low to resolve atomic-level features, the cholesterol-free liposomes were unable to elicit a similar interaction between the surface and C3 – strongly suggesting cholesterol is crucial for such an interface.

Based on these observations, we propose a non-proteolytic activation mechanism of native C3 making C3 a specific recognition molecule of altered self that takes place on scrambled cell membranes. The mechanism allows focused deposition of large amounts of C3(H_2_O) molecules on cell membranes, thereby initiating and fueling the AP amplification loop. It is also a shortcut to opsonize scrambled cell membranes without proteolytic cleavage of C3 and without generating inflammatory peptides such as C3a. Scrambling of cell membranes exposing phosphatidyl serine is a general non-self signal that operates in platelet activation, apoptosis, necroptosis, hematological disorder, neuron synaptic pruning etc (46–48) and may reflect the activation mechanism of C3 that is available already in evolutionary old species e.g. tunicate (500-million-year-old) in which other complement factors, such as FB and FD, are absent and where C3 is part of extracellular phagocytic and intracellular processes (49).

## Data Availability

The original contributions presented in the study are included in the article/supplementary material. Further inquiries can be directed to the corresponding author.
